# Workplace incivility experienced by foreign national nurses in a Saudi Arabian hospital

**DOI:** 10.4102/hsag.v31i0.3237

**Published:** 2026-03-11

**Authors:** Livhuwani S. Lavhengwa, Nellie Naranjee, Krishnavellie Chetty

**Affiliations:** 1 Department of Nursing, Faculty of Health Sciences, Durban University of Technology, Durban, South Africa

**Keywords:** workplace incivility, foreign national nurses, organisational culture, Saudi Arabia, military hospital, nurse well-being, patient safety, safe workplace

## Abstract

**Background:**

Workplace incivility is a persistent challenge in nursing. In Saudi Arabia, where foreign national nurses constitute a large part of the healthcare workforce, particularly in military hospitals, there is limited research on their experiences with workplace incivility and its effect on their well-being and patient care.

**Aim:**

This study explored the factors influencing workplace incivility experienced by foreign national nurses in a Saudi Arabian military hospital and examined how these experiences impact their personal and professional well-being.

**Setting:**

This study was conducted in the Southern Region of Saudi Arabia, in the province of Aseer. The study setting was the medical and surgical units in a military hospital.

**Methods:**

A qualitative design using semi-structured interviews was employed with foreign national nurses. Purposive sampling ensured diverse representation across nationality, experience and clinical areas. Thematic analysis identified patterns, contributing factors, and perceived consequences of workplace incivility.

**Results:**

Four subthemes emerged under organisational factors affecting workforce incivility and job satisfaction: ineffective leadership styles, discrimination among employees, poor employee engagement and empowerment, and lack of support from managers.

**Conclusion:**

This study highlights the distinct challenges foreign national nurses face in Saudi Arabia’s healthcare context. The study contributes to evidence-based strategies for creating respectful, inclusive, and psychologically safe workplaces. The findings will inform hospital policies and support Saudi Arabia’s Vision 2030 healthcare reforms.

**Contribution:**

By amplifying the voices of foreign national nurses, the study sheds light on socio-cultural, organisational, and legal factors influencing workplace incivility. Aligning with Vision 2030, the study offers practical guidance for fostering inclusive workplaces and enriches theoretical and methodological understanding of incivility in multicultural healthcare settings.

## Introduction

The healthcare sector of the Kingdom of Saudi Arabia is in a phase of substantial transformation aligned with Vision 2030, which aims to improve healthcare service delivery, workforce development, and patient care outcomes. Central to achieving these goals is a diverse and multinational nursing workforce, with a significant proportion of foreign national nurses employed across both public and private healthcare sectors. However, despite their invaluable contributions, foreign nurses often encounter considerable workplace challenges, with workplace incivility emerging as a critical and under-addressed issue. Workplace incivility, the term used to describe workplace behaviour that is rude, disruptive, and disrespectful and that promotes discord and disharmony (Jackson, Usher & Cleary 2024), is a growing global concern in healthcare. Workplace incivility can include a range of negative interpersonal behaviours such as dismissiveness, aggression, condescension, exclusion, rudeness, blaming, accusing, sabotaging, unhelpfulness, micromanagement, disregarding personal boundaries, and undermining (Wilson, Urban & Smith 2023). Incivility is prevalent in nursing across an array of contexts and situations. High levels of experience of workplace incivility have been extensively described across the profession in students of nursing, newly graduated nurses, clinical nurses, and nurse academics (Blackstock et al. 2022; Clark, Landis & Barbosa-Leiker 2020; Singh et al. 2020; Wilson et al. 2023). For foreign national nurses, these experiences are often intensified by factors such as cultural differences, language barriers, hierarchical structures, and limited institutional protection.

In the Saudi context, the traditional and hierarchical or male-dominated structures of some healthcare institutions, combined with the Kafala sponsorship system, may further exacerbate the vulnerability of foreign healthcare workers. A culture that tolerates incivility can lead to emotional exhaustion, job dissatisfaction, and increased staff turnover, all of which threaten the stability and sustainability of healthcare service delivery (Loh & Saleh 2024). This study holds social value by giving voice to foreign national nurses and highlighting the social injustices and systemic neglect embedded in incivility. By identifying the factors contributing to this behaviour, the research aims to promote ethical work environments, emotional well-being, and a culture of civility that enhances quality care for all.

While the issue of workplace incivility has been extensively explored in Western healthcare settings, there is a notable gap in empirical research focusing on the lived experiences of foreign national nurses in the Middle East, particularly in Saudi Arabia. Limited studies have investigated how socio-cultural and organisational dynamics influence the incidence and impact of workplace incivility in these settings. Given the high percentage of expatriate nurses in Saudi Arabia, accounting for 40% of public sector and 94.5% of private sector nursing staff (Ministry of Health [MOH] Saudi Arabia 2018), it is imperative to understand how incivility manifests in this context. The scientific originality of this study lies in its examination of how foreign nurses perceive and experience incivility, and how cultural, legal, and organisational structures shape these interactions.

Moreover, this study differentiates itself by examining workplace incivility not only as a personal grievance but also as a systemic and structural issue that influences organisational culture, healthcare quality, and staff retention. The study contributes to a relatively under-researched field and offers evidence-based recommendations for leadership, human resources, and policymakers to develop culturally responsive and inclusive workplace interventions.

Workplace incivility, marked by subtle and low-intensity behaviours with ambiguous intent to harm or violate mutual respect, can significantly disrupt professional environments. In healthcare, such behaviour compromises not only employee morale but also patient safety. Incivility creates a toxic work culture that fosters hostility, impedes collaboration, and undermines team performance (Schilpzand, De Pater & Erez 2016). Despite the prevalence of incivility in nursing, many healthcare providers continue to tolerate or ignore these behaviours as a result of fear of retaliation, lack of awareness, and inadequate institutional policies (Alsadaan, Ramadan & Alqahtani 2024).

Foreign national nurses are particularly vulnerable to workplace incivility as a result of factors such as language barriers, cultural misunderstandings, limited social support, and structural power imbalances. In Saudi Arabia, where a significant proportion of the nursing workforce comprises foreign national nurses, particularly from the Philippines, India, and Egypt, these issues are pronounced. The hierarchical organisational culture and the Kafala sponsorship system may further limit these nurses’ ability to report or address mistreatment (Alshehry et al. 2019). Saudi Arabia officially abolished the Kafala sponsorship system in June 2025, ending a decades-old framework that tied migrant workers’ legal status to their employers. Under the old system, employers controlled workers’ job changes, travel, and residency, often leading to exploitation and restricted freedoms. The new contractual labour system now allows migrant workers to change employers freely, leave the country without exit visas, and access clearer legal protections. The reform affects about 13 million foreign workers and aims to reduce human rights abuses and improve labour mobility (Economic Times 2025).

The need for such research is further emphasised by Saudi Arabia’s Vision 2030, which calls for healthcare improvements and a more inclusive and efficient workforce (Ministry of Health Saudi Arabia 2018). Yet, to date, no extensive study has examined the experiences of foreign national nurses with workplace incivility in the Saudi Arabian context. Incivility not only impacts the emotional and professional well-being of nurses but also contributes to increased staff turnover, reduced patient satisfaction, and diminished quality of care (Khan, Elahi & Abid 2021).

The prevalence and economic impact of workplace incivility are profound and cannot be overlooked. The impact of workplace incivility on foreign nurses from diverse cultural backgrounds remains underexplored, with limited insights available from previous studies. This gap in the literature is particularly relevant as Saudi Arabia embraces Vision 2030, its ambitious strategy for economic diversification and social reform. Aligned with this vision, the MOH is committed to enhancing healthcare services for all citizens, and notable progress has already been achieved (Ministry of Health Saudi Arabia 2018).

Nurses constitute the largest segment of the healthcare workforce in Saudi Arabia (Chetty, Naidoo & Naranjee 2021). Workplace violence and incivility towards nurses pose serious challenges for hospital administrators, often resulting in negative consequences such as diminished performance, decreased self-confidence, and increased staff turnover (Alkorashy & Al Moalad 2016). The MOH announced a significant increase in the number of nurses registered with the Saudi Commission for Health Specialities, marking this achievement on International Nurses Day. According to a report from the Ministry of Health (2024), the total number of nurses in 2023 reached 235 461, employed across both the public and the private sectors. This growth forms part of the Health Sector Transformation Programme, one of the key initiatives under Saudi Vision 2030, which aims to support, empower, and ensure sufficient staffing and high-quality healthcare services. Between 2016 and 2023, the number of male and female nurses increased by more than 23%. Currently, the MOH employs over 106 000 nurses, around 15 000 serve in other government agencies, and the private sector employs approximately 67 000 nurses. Of the total, 94 021 are Saudi nationals (General Authority for Statistics 2025). Foreign national nurses comprise about 70% of the total number of nurses and are mostly Indian, Filipino, and Malaysian (Ministry of Health Saudi Arabia 2018). Given their significant presence in the healthcare system, it is both timely and necessary to examine the experiences of foreign national nurses in Saudi Arabia, particularly in relation to workplace incivility and its broader implications. When incivility is tolerated within organisational culture, it opens the door to other unacceptable behaviours, making the work environment unsafe.

This article aims to foster a supportive environment for employees, whereby co-workers, leaders and various organisations can provide and receive emotional support. Employees who perceive a high socially supportive climate feel comfortable reporting incivility and feel confident in receiving support, protection, and redress (Tong, Chong & Johnson 2019). For example, they expect co-workers to condemn or expect organisational authorities to penalise perpetrators. The findings from this article will create awareness among potential perpetrators, deterring uncivil behaviour towards these employees as a result of the anticipated negative consequences stemming from organisational or social sanctions. These findings will inform policymakers, managers, and relevant stakeholders to re-establish laws and legislation within the organisation or establish norms against severe and explicit mistreatment in the workplace.

This study aims to explore the factors influencing workplace incivility experienced by foreign national nurses in a Saudi Arabian military hospital. Furthermore, the study investigates how such experiences affect the personal and professional well-being of foreign national nurses. Ultimately, it aims to generate evidence-based strategies for promoting a culture of respect, inclusivity, and civility in Saudi healthcare institutions.

## Research design and methods

This study adopted a qualitative, exploratory phenomenological design to examine the lived experiences of foreign national nurses regarding workplace incivility in a Saudi Arabian hospital. This approach was chosen for its capacity to explore participants’ perceptions in depth, allowing the phenomenon to emerge through open and subjective engagement during interviews.

Phenomenology, as a research methodology, focuses on uncovering the shared meanings and patterns of participants’ experiences, rather than individual variations. It emphasises authenticity, openness, and the researcher’s willingness to understand the phenomenon without bias. According to Polit and Beck (2022), key principles such as immediacy, encounter, and meaning are essential for capturing the true essence of lived experience. As such, the study aimed to identify the core themes and collective meanings behind foreign nurses’ experiences of incivility in the workplace, contributing to a deeper understanding of this social phenomenon within the Saudi healthcare context.

### Research setting

The military hospital in the Southern Region of Saudi Arabia is the designated hospital for the treatment of patients from military families, employing a total of 1200 nurses, 80% of whom are foreign nationals. The General Military Hospital provides 100% sponsored healthcare for its patients and operates 520 beds across all medical specialities, including Intensive Care Unit, Critical Care Unit, paediatrics, operating rooms, oncology, and dialysis. The hospital is situated in the Southern Region of Saudi Arabia in the province of Aseer.

### Population and sample

The study focused on a target population of 50 foreign national nurses employed in the medical and surgical units of a hospital in Saudi Arabia. From India, South Africa, and the Philippines, 18 nurses were selected based on their direct involvement in patient care, using purposive sampling. Three participants were involved in the pilot study, and 12 took part in the main study. Although data saturation was achieved after the 10th interview, two additional participants were included to ensure the completeness and confirmability of the data. This step was taken to verify that no new themes would emerge and to enhance the trustworthiness, credibility, and dependability of the study findings. Including the two extra participants provided further confidence that the data accurately reflected participants’ experiences and that the themes were well developed and comprehensive.

### Data collection

The data collection method was qualitative and consisted of individual one-on-one semi-structured interviews with the participants. The main feature of the interviews was to facilitate the interviewees to share their perspectives and experience related to the culture of workplace incivility experienced by foreign national nurses while caring for medical and surgical patients in the Saudi Arabian Hospital under study. According to Gray and Grove (2020), a semi-structured interview is an in-depth interviewing approach designed to uncover complex meanings and interpretations that are not easily accessible through positivist research methods. The medical and surgical units and participants were coded to ensure anonymity. The interview questions were designed to address the research question, which aimed to explore the various factors influencing incivility in the workplace among foreign national nurses in a Saudi Arabian hospital.

For the face-to-face interviews, all coronavirus disease 2019 (COVID-19) safety measures were strictly observed. These included maintaining a 1.5-metre distance between the interviewer and the participant, regular hand sanitisation, and the use of face masks (Chetty et al. 2024). The interviews happened from 25 May 2023 to 27 May 2023. They were conducted in a private room. They lasted from 30 min to 45 min and were conducted in English, as all participants spoke and understood English. The venue was set up by the researcher in advance for the scheduled interviews, ensuring that the environment was conducive for the interviews and that the participants would feel comfortable and confident that their privacy, anonymity, and confidentiality were maintained. The individual semi-structured interviews allowed the foreign national nurses to share their personal views and experiences without fear of intimidation by the rest of the group. Permission to record the interviews was granted by the participants.

### Data analysis

A qualitative, inductive approach was used to analyse the data, aligning with the study’s objectives and interview questions. Data collection and analysis occurred concurrently. Interviews were transcribed within 48 h, verified for accuracy, and supplemented with field notes. Creswell and Creswell’s (2018) six-step framework guided the process:

- Organising and preparing data
- Reading through all the data
- Manual coding and secure storage of data
- Generating descriptions, themes, and subthemes
- Interrelating themes
- Constructing the final report

Data were analysed by using manual coding, a systematic process in which qualitative data were examined, segmented, and categorised by the researcher without the aid of software. Manual coding involved reading through transcripts and field notes repeatedly to identify meaningful patterns, concepts, and recurring ideas. Each segment of data was assigned a code that represents its underlying meaning, allowing themes and subthemes to emerge organically from the data.

### Data interpretation

Data interpretation involved drawing inferences and meta-inferences from the analysed findings to address the study’s aims, objectives, and research questions. This activity helped identify patterns, meanings, and conclusions relevant to the phenomenon under study.

Data were securely stored in both hard and electronic formats. Hard copies were kept in a locked cabinet, and electronic files were password-protected. Voice recordings and field notes were used to ensure accurate data capture. All ethical protocols for data confidentiality and storage were strictly followed. Data will be retained for five years, after which it will be securely destroyed.

### Researcher reflexivity

Reflexivity was maintained throughout the data collection and analysis processes to minimise researcher bias and enhance the trustworthiness of the findings. The researcher continuously reflected on personal assumptions, professional background, and potential influences on data interpretation through the use of reflexive journaling and field notes. Regular debriefing sessions with the research supervisor and peer discussions were conducted to ensure objectivity and to validate the interpretation of emerging themes. This reflective practice allowed for a transparent and balanced representation of participants’ perspectives.

### Trustworthiness of the study

Lincoln and Guba’s (1985) four criteria were applied to ensure the trustworthiness of this qualitative study:

Credibility was ensured through purposive sampling, prolonged engagement, and data saturation. The researcher’s accurate interpretation and presentation of participants’ views enhanced the truthfulness of the findings. Transferability was achieved through thick descriptions and purposive sampling, allowing the findings to be applied to similar settings or populations. Dependability was supported by a detailed account of data collection and analysis methods. A pre-test with three individuals outside the main study enhanced consistency. Confirmability was ensured by using audio recordings and field notes to reduce researcher bias. All materials were preserved for potential auditing. Generalisability was addressed by collecting and analysing data in depth, allowing comparison with similar studies and broader application. Authenticity was reflected through honest representation of participants’ emotions and experiences, particularly regarding incivility towards foreign national nurses. Audio recordings were used for accuracy and will be discarded post-data analysis.

In addition to adhering to Lincoln and Guba’s framework of credibility, dependability, confirmability, and transferability, further strategies were employed to strengthen the trustworthiness of the study. Member checking was conducted by sharing summaries of the interpreted data and emerging themes with selected participants to confirm the accuracy and authenticity of their perspectives. Peer debriefing sessions with supervisors were also held to critically review the coding process, theme development, and interpretation of findings. These engagements provided external perspectives, reducing potential researcher bias and enhancing the credibility and confirmability of the results. Maintaining a detailed audit trail further ensured transparency and methodological rigour.

### Ethical considerations

Ethical clearance to conduct this study was obtained from the Durban University of Technology’s Institutional Research Ethics Committee (No. 180/30). This study adhered to rigorous ethical standards to ensure the protection and dignity of all participants. Gatekeeper permission was sought and obtained from the Ethics Committee of the participating hospital to conduct research at the proposed study site.

Informed consent was a fundamental principle, with participants fully briefed on the purpose, procedures, potential risks, and their rights regarding the study. Participation was voluntary, and individuals were free to withdraw at any point without facing any penalties. Written consent was obtained, witnessed, and countersigned by the researcher after providing comprehensive information. Confidentiality and anonymity were strictly maintained. Participants’ identities were protected by separating consent forms from survey data and interview guides. Data were securely stored in locked cabinets and password-protected electronic systems, accessible only to the researcher and supervisors. All interviews were conducted privately, and only non-identifiable information, such as the department and designation, was used for data analysis. All physical and digital data will be destroyed after five years, in line with institutional policy.

The principles of beneficence and non-maleficence guided the researcher to act in the best interest of the participants, ensuring their well-being and minimising any risk of harm: physical, emotional, or otherwise. Participants were not coerced or forced to participate in the study and were reassured that their involvement posed no risk. They were allowed to ask questions and were encouraged to participate only if they were comfortable. Respect for participants was upheld throughout the data collection process. Their right to self-determination was maintained by avoiding deception, coercion, or the offer of excessive incentives. Privacy was a priority, and data were treated with strict confidentiality, ensuring no link between the shared information and participant identity. Overall, the study maintained a high standard of ethical integrity in line with professional, legal, and social research obligations.

## Results

One major theme and four sub-themes were derived from the objective that sought to determine the factors influencing incivility in the workplace, as per Table 1.

**TABLE 1:** Themes and sub-themes.

Objective	Theme	Sub-theme
To determine the factors influencing incivility experienced by foreign national nurses in the workplace.	Theme 1: Organisational factors influencing workplace incivility and job satisfaction	1.1 Ineffective leadership styles 1.2 Discrimination among employees 1.3 Poor employee engagement and empowerment 1.4 Lack of support from managers

### Characteristics of the study participants

A total of 12 participants, who were foreign national nursing staff, were interviewed from the medical and surgical units of the selected hospital. Ten of the participants were women, and two were men. Three of the participants were between the ages of 41 and 50, four were between 31 and 40, and five were between the ages of 21 and 30.

The experience levels measured in the years of service in the nursing profession ranged as follows: Five participants had between three and five years of experience, four had between five and 10 years of experience, and three participants had between 11 and 20 years of experience. The participants were selected from the designated medical and surgical units, comprising adult male and female patients, to better understand the nurses’ experiences of workplace incivility in a Saudi Arabian hospital, in a wider context. The findings also guided recommendations for creating a managerial support framework that outlines key motivational, behavioural, and environmental influences, as well as the human needs and opportunities for professional development relevant to nurses working in medical and surgical units (Chetty et al. 2024). Among the 12 participants, one held a Diploma in Nursing Science, while the remaining 11 had Bachelor’s Degrees in Nursing. In addition to these basic qualifications, four participants had further postgraduate credentials, including a Master’s Degree in Nursing and a Master’s Degree in Medical and Surgical Care Nursing Science.

### Emergent themes

In this article, the themes and subthemes are illustrated with verbatim quotations from participants to reinforce their significance within the results. All interviews with foreign national nurses in the medical and surgical units were conducted in English, as it is the language used in the workplace. The excerpts included in this article are drawn directly from the original interview transcripts. Only minor edits, such as the addition of punctuation marks like full stops, commas, and question marks, were made to enhance clarity and readability. This approach enabled the researcher to convey participants’ accounts accurately while preserving the authenticity and integrity of the data (Chetty et al. 2024).

#### Theme 1: Organisational factors influencing workplace incivility and job satisfaction

When participants were asked about the factors contributing to workplace incivility, several expressed experiencing unconscious biases that led to psychological distress, increased job withdrawal, reduced job satisfaction, and in some cases, symptoms of depression. Participants highlighted that highly stressful job conditions often culminate in emotional exhaustion, further diminishing their satisfaction with their roles. Many described workplace incivility as a significant psychological stressor that affected them both emotionally and cognitively, ultimately impacting their overall well-being. These experiences collectively contributed to a decline in job satisfaction. From the interviews, four subthemes emerged, which are discussed below:

**Sub-theme 1.1: Ineffective leadership styles:** During probing, the participants were asked how ineffective leadership styles influence their morale and job satisfaction. Most (10) of the participants expressed that they felt like leaving their jobs, their morale was low, and this was influencing their job satisfaction in their units. Participants further expressed that such managers and/or supervisors affected their quality of patient care through various acts, such as not recognising all extra efforts which the staff took to ensure that normal workflow was maintained. Some of the line managers showed a level of favouritism in the way they led the department, which drained the morale of other good performers. This effect is evident in the following excerpts:

‘On most of my days at work, in the medical ward … I just feel like taking my bag and leaving that place because there is nothing which I do that makes my shift leader happy or proud. Instead, she would go ahead and praise everyone else for minor tasks and turn a blind eye when it was time to acknowledge my work. It’s as if she wanted me to feel that I did not belong in that team.’ (Participant 2, Woman, Age 50)

‘I love my job. I enjoy working with my patients … at times, not always, I feel overworked, and my charge nurse bullies me and threatens me that she will not do my performance appraisals to punish me if I complain to management about anything in the ward … at times, not always.’ (Participant 5, Woman, Age 30)

**Sub-theme 1.2: Discrimination among employees:** Putting discrimination into the context of employees and the workplace specifically increases perceived organisational dehumanisation, which in turn increases participants’ self-objectification. Self-objectification is associated with lower job satisfaction and dignity at work (Sainz, Moreno-Bella & Torres-Vega 2023). When employees feel like they are not being treated well because of certain elements of discrimination, they may start to feel resentful towards the management team and other employees who seem to benefit from the discriminatory acts. This negative attitude has a snowballing effect on the overall performance of the staff, department, and hospital at large.

Based on the feedback from the participants, discrimination in the workplace has proven to be a driving force for incivility and general poor performance of staff or teams. In addition, there is a statistically significant negative correlation between intolerance for discrimination affecting job-related elements (Hossny & Sabra 2020). Multiple studies have discovered a correlation between low rates of retention as a direct impact of workplace incivility as a result of discrimination. The following statements attest to the above:

‘My team leader has a discriminatory behaviour towards me, and she is not fair to me … I can see that because I am not originally from here. She does not like me. Then she gives me eight patients to care for. Those who are from here will only take two patients. When I questioned her actions, she then threatened me about my job and indicated that if I could not cope, I must leave the job. I need her to understand that at times I can take more patients, but at times I need a break, and all nurses are here for the same reason, regardless of where we migrated from; therefore, why must I suffer alone? I sometimes feel she is victimising me because I am from a different nationality, and she does not like me.’ (Participant 4, Woman, Age 40)

‘My manager is sometimes fair and unfair, especially when it comes to leave allocation and approvals. I asked to go home and be with my family because my father was ill. My head nurse refused because I just came back from my vacation. This situation was out of my control, and I needed the leave … she said that I demand, and I do not have respect. I am emotional due to that experience, and I do not feel a sense of job satisfaction. This affected my work, and I was constantly making mistakes … I requested to move to another department just for peace because I sensed she and other staff in the unit wanted to find fault in my work … but I tried my best to perform well.’ (Participant 6, Woman, Age 25)

**Sub-theme 1.3: Poor employee engagement and empowerment:** High-performance work systems can be moderately negative towards the employee if there are no adequate employee engagement and empowerment platforms in place. Nurses, who make up the largest proportion of the healthcare workforce, often face numerous workplace pressures, including extended working hours, staff shortages, and the emotional impact of patient deaths (Saedpanah et al. 2022). Nurses are constantly dealing with stress arising from their job or workplace. Therefore, to ensure that they are always on top of their game, there must be a serious commitment from management to ensure sustainable employee engagement and empowerment programmes.

By putting sustainable engagement and empowerment programmes in place, it will be easier for the nurses to convert all the daily stressors into a daily, constructive coping mechanism. There are many types of stress, such as technical stress, managerial stress, mental stress, and burnout stress (Kapur 2021). It may arise in every kind of job. Job stress refers to physical and emotional responses that occur when the requirements of the job do not match the capabilities, resources, or needs of the worker (Soni & Kumar 2022). Most of the participants expressed that they could manage the workload presented to them, but if only there was some effort from management to empower them with ways to handle the job stress. This view is stated in the following excerpts:

‘I have been working in this department for more than three years … and now things are changing … work stresses are increasing by the day, we have been waiting and hoping that our managers will meet us halfway through some empowerment or engagement campaigns, to help us deal with all of this. Team members that do not contribute are stressing us out in the ward, and only if we had a decent platform to engage, we would have addressed this a long time ago.’ (Participant 3, Woman, Age 35)

‘As a foreigner, I give my best … I came to Saudi to make money for my family … I do not get job satisfaction … the patients are very rude and aggressive and treat us foreigners with no respect. Some doctors also have no respect … they shout at us and speak Arabic, saying bad words. They think we do not understand but we do … yes, I get very stressed when doctors are speaking bad words, especially the Egyptian doctors. They have no respect for us nurses. I really wish management would set up something for us to communicate and engage with them and to also impose certain rules and boundaries for the doctors to respect us that would really empower us a lot.’ (Participant 7, Woman, Age 28)

**Sub-theme 1.4**: **Lack of support from managers:** Workplace incivility can be best explained with the help of the incivility continuum, which ranges from negative behaviour to physical aggression. Paul (2021) states that discourteous behaviours can be characterised as rude and discourteous, displaying disrespect for others. Victims of these unpleasant acts are subjected to various characteristics of workplace incivility. Incivility is generally marked by subtle violations of organisational and interpersonal norms, in which the behaviour deviates from expected standards of respect and professionalism. These actions often carry an ambiguous intent, making it unclear whether the perpetrator aims to cause harm, which complicates the victim’s ability to respond or report the behaviour. Although the behaviours are typically low in intensity, such as dismissive comments, exclusion, eye-rolling, or lack of acknowledgement, they accumulate over time and create a hostile, stressful, and psychologically unsafe work environment. This persistent exposure can negatively affect victims’ emotional well-being, job satisfaction, and overall sense of belonging in the workplace (Kim & Lee 2024). Incivility can be instigated by various means and become a part of one’s life. Workplace incivility is a growing concern for many organisations, as it results in toxic work environments. Incivility at work affects the psychological well-being of employees in a negative manner and thus influences their work performance. It is evident on taking this and the context into consideration that a line manager and/or supervisor and/or shift leader must be present and ensure that they render support to those who might be experiencing signs of incivility:

‘I sometimes feel like some of our shift leaders take sides in a way. You can find that, maybe, when I, for example, go report something which offended me or belittled me, they will tell me that they will look into the matter and get back to me, but then days and weeks will go by with no one coming back and giving me feedback, but when a local nurse raises a concern; staff meetings can even be held to address the situation same time. So, I don’t know if anyone else has felt the same way about this.’ (Participant 8, Woman, Age 24)

‘As the foreign nurses, we sometimes feel like our concerns and complaints are not handled with urgency until a big problem comes up. Then you will see management quickly trying to make some damage control and fix the problem. I am not sure if this has always been this way before I started working here, but yes, it’s a problem, and we are not happy about it, I must say.’ (Participant 3, Woman, Age 35)

The findings show that behavioural, environmental, emotional, and psychosocial factors influenced the foreign national nurse’s work performance both negatively and positively because of various incivility factors such as bullying in the workplace, harassment, verbal abuse, and emotional abuse with victimisation. Creating a positive working environment and showing respect to nurses are the key to combatting a feeling of disrespect and low self-esteem. Leaders should focus on developing employee well-being programmes and adhering to the incivility and behavioural misconduct legislation and policies in Saudi Arabia.

## Discussion

The findings from this study reveal that workplace incivility in healthcare settings, particularly among foreign nurses, is a multidimensional issue that significantly impacts morale, job satisfaction, psychological well-being, and overall productivity. Four interrelated subthemes were identified, each offering insight into how organisational dynamics and interpersonal relationships contribute to the perpetuation of incivility and its consequences.

Ineffective leadership styles emerged as a core issue, with participants describing a lack of recognition, favouritism, and emotionally unsupportive behaviours from their supervisors. These findings are consistent with studies that have shown how poor leadership contributes to disengagement, emotional fatigue, and increased turnover intentions (Thompson et al.^2024^). When staff perceive their efforts as being ignored or unfairly evaluated, that perception undermines their sense of belonging and leads to job dissatisfaction.

Closely linked to leadership issues is discrimination among employees, which was evident in participants’ descriptions of differential treatment based on nationality, language, or educational background. Discrimination fosters resentment, reduces team cohesion, and intensifies feelings of exclusion. Sainz et al. (2023) argue that perceived organisational dehumanisation and self-objectification are closely associated with experiences of workplace discrimination. In Hossny and Sabra’s (2020) study, foreign nurses consistently felt as if they were marginalised, overburdened, and denied fair treatment, factors known to negatively influence retention and psychological safety.

The theme of poor employee engagement and lack of empowerment revealed that many nurses felt voiceless, overworked, and excluded from decision-making processes. Without proper channels for communication and engagement, staff are left to internalise stress, leading to emotional exhaustion and poor performance. The absence of mechanisms for feedback or support further isolates employees and reinforces hierarchical power dynamics (Walker 2024). Participants longed for structured empowerment programmes and meaningful platforms to express concerns, which could mitigate burnout and strengthen team dynamics.

Finally, lack of managerial support was a recurring concern. Participants described how their complaints were dismissed or ignored, especially when compared to how swiftly concerns of local staff were addressed. This selective responsiveness erodes trust and perpetuates perceptions of injustice. Agarwal, Singh and Cooke (2023) note that incivility exists on a continuum and, when left unaddressed by leadership, can escalate from subtle forms of disrespect to more overt forms of aggression. The emotional and psychological toll of this neglect was apparent in participants’ narratives, with several considering leaving their positions as a result of feeling undervalued or unheard.

Foreign national nurses should be valued for their tireless commitment to patient care and be motivated with ongoing appreciation, which plays a vital role in a nurse’s productivity. Collectively, these themes suggest that workplace incivility is not an isolated occurrence but rather a systemic issue deeply embedded in leadership practices, organisational culture, and interpersonal relationships. The findings call for a holistic organisational response that includes leadership training, anti-discrimination policies, fair workload distribution, and the establishment of safe, responsive communication channels for all staff, regardless of nationality or status.

The study applied Weiss and Cropanzano’s Affective Events Theory (AET) to interpret how workplace incivility influences the emotions, attitudes, and performance of foreign national nurses in a Saudi hospital. Affective Events Theory explains that workplace events trigger affective reactions that shape job satisfaction, commitment, and behaviour. The findings indicate that repeated exposure to rudeness and lack of organisational support generate negative emotions that undermine well-being and productivity. Applying AET provided a valuable framework to link emotional experiences with behavioural outcomes, offering insights for policies and interventions that promote a respectful and supportive work environment.

### Recommendations

The findings revealed several organisational factors influencing workplace incivility and job satisfaction among foreign national nurses. Based on these findings, the study recommends targeted strategies aligned with the identified subthemes to promote civility, improve job satisfaction, and introduce policy reforms.

#### Implement leadership development programmes

Ineffective leadership was identified as a key contributor to workplace incivility. To address this issue, healthcare institutions should implement leadership development programmes focusing on emotional intelligence, transformational leadership, and effective communication. Such initiatives will equip leaders to foster respect, civility, and supportive team dynamics. Regular supervisory check-ins, constructive feedback mechanisms, and mentorship opportunities should be incorporated to reinforce positive leadership practices.

#### Implement policy reforms

Discrimination emerged as a significant concern affecting morale and workplace harmony. Organisations should develop and enforce robust diversity and inclusion policies, complemented by cultural competence training for all staff. Awareness campaigns and reflective workshops can help nurses understand the impact of discriminatory behaviours, recognise biases, and promote fairness and equity in daily interactions.

#### Promote participatory decision-making

Low engagement and lack of empowerment were linked to dissatisfaction and increased incivility. Organisations should promote participatory decision-making, professional development opportunities, and recognition programmes. Departmental workshops and regular team meetings can provide platforms for nurses to share experiences, discuss challenges, and contribute ideas, fostering a sense of ownership and collective responsibility.

#### Provide mechanisms for addressing incivility incidents

The absence of managerial support was identified as a key factor in workplace incivility. Establishing peer support systems, open communication channels, and rapid response teams for addressing incidents of incivility can enhance both emotional and professional well-being. Additionally, reflective and self-awareness training can help nurses manage stress, develop empathy, and contribute to a supportive and civil work environment.

By implementing these recommendations, healthcare institutions can develop a structured managerial support framework that enhances civility, improves job satisfaction, and strengthens professional growth among foreign national nurses. These strategies align with the goals of Vision 2030 by promoting workforce empowerment, equitable access to professional development, and the creation of resilient, high-performing healthcare teams that deliver quality patient care.

#### Strengths

This study’s focus on foreign national nurses in Saudi Arabian military hospitals addresses a gap in the literature, offering unique insights into an under-researched healthcare setting. Using qualitative semi-structured interviews allowed for in-depth exploration of participants’ experiences, capturing workplace incivility from their perspective. Purposive sampling ensured diverse representation across nationalities, clinical areas, and experience levels, enhancing the transferability of findings. Rigorous thematic analysis identified organisational, cultural, and managerial factors contributing to incivility, supporting the development of practical interventions. The findings are directly relevant to hospital administrators and policymakers, aligning with Saudi Arabia’s Vision 2030 healthcare transformation goals, while amplifying the voices of an often-overlooked group.

#### Limitations

The study was conducted with a limited number of participants from a single setting, which may not represent nurses in other regions or institutions. Its findings are contextually bound, limiting generalisability. Future research should include larger, more diverse samples across multiple healthcare settings and cultural contexts, and consider longitudinal designs to track changes in workplace culture and perceptions over time.

#### Areas for future research

This study offers valuable insights that can inform the development of future interventions aimed at fostering civility and respect within healthcare environments. Several critical areas warrant further exploration to strengthen organisational strategies and promote a culture of inclusivity and psychological safety.

Firstly, evaluating organisational culture remains imperative. Future studies should investigate how incivility is tolerated, normalised, or unintentionally reinforced within various departments or units. Such investigations will facilitate the design of culture-specific interventions that address entrenched behavioural norms and organisational dynamics. Secondly, research should examine the barriers to the success of existing civility interventions. Understanding why certain interventions fail or succeed can lead to the creation of innovative, context-sensitive strategies that are both effective and sustainable. Cultural considerations further complicate the manifestation of incivility. Comparative studies across collectivist and individualist cultural contexts are needed to inform culturally responsive and adaptable interventions, especially in diverse or multicultural healthcare teams.

In addition, leadership responses to incivility merit further scrutiny. The way leaders handle incidents of incivility can have a lasting impact on team morale, cohesion, and perceptions of justice within the workplace. Research should assess the long-term effects of leadership approaches on organisational climate and employee outcomes. Future research should also compare the prevalence and nature of workplace incivility across different healthcare settings, including public and private sectors, and rural and urban hospitals. These comparative studies will help identify context-specific factors that influence the frequency and type of incivility encountered by nurses. Longitudinal research is recommended to assess the extended impact of workplace incivility on nurses’ mental health, career trajectories, absenteeism, and retention within the profession.

Lastly, further studies should explore how nurse managers and senior leaders perceive and respond to incivility. This research includes their role in prevention, conflict resolution, and the cultivation of a positive organisational culture.

By addressing these areas, future research can contribute to evidence-based strategies that not only enhance the well-being of healthcare professionals but also improve the quality of care and safety of patients. A sustained focus on civility in healthcare workplaces is essential for building resilient, respectful, and high-performing clinical environments.

## Conclusion

The findings of this study highlight the multifaceted and deeply rooted nature of workplace incivility within the nursing profession. Participants consistently linked incivility to ineffective leadership, lack of managerial support, perceived discrimination, and increasing workload demands, all of which negatively impacted job satisfaction and overall well-being. The psychological and emotional burden of incivility has led many nurses to experience feelings of exclusion, stress, and burnout, often prompting thoughts of leaving the profession. These experiences were further intensified by the residual effects of the COVID-19 pandemic, which exacerbated both professional and personal challenges. The insights gained from this study emphasise the urgent need for healthcare institutions to develop more inclusive, supportive, and responsive leadership strategies, foster positive work environments, and address systemic inequities to enhance nurse retention, satisfaction, and patient care outcomes.
